# Challenges to the broad application of allogeneic natural killer cell immunotherapy of cancer

**DOI:** 10.1186/s13287-022-02769-4

**Published:** 2022-04-12

**Authors:** Philippa R. Kennedy, Martin Felices, Jeffrey S. Miller

**Affiliations:** Division of Hematology, Oncology and Transplantation, Department of Medicine, University of Minnesota, MCRB Rm 520, 425 E River Rd Parkway, Minneapolis, MN 55455 USA

**Keywords:** Adoptive, Allogeneic natural killer cell, Immunotherapy, Metabolism, Hypoxia, Penetrance persistence

## Abstract

Natural killer (NK) cells are innate immune cells that recognize malignant cells through a wide array of germline-encoded receptors. Triggering of activating receptors results in cytotoxicity and broad immune system activation. The former is achieved through release of cytotoxic granules and presentation of death receptor ligands, while the latter is mediated by inflammatory cytokines, such as interferon-γ and tumor necrosis factor α. Early success with ex vivo activation of NK cells and adoptive transfer suggest they are a safe therapeutic with promising responses in advanced hematologic malignancies. In particular, adoptive NK cell therapies can serve as a ‘bridge’ to potentially curative allogeneic stem cell transplantation. In addition, strategies are being developed that expand large numbers of cells from limited starting material and mature NK cells from precursors. Together, these make ‘off-the-shelf’ NK cells possible to treat a wide range of cancers. Research efforts have focused on creating a range of tools that increase targeting of therapeutic NK cells toward cancer—from therapeutic antibodies that drive antibody-dependent cellular cytotoxicity, to chimeric antigen receptors. As these novel therapies start to show promise in clinical trials, the field is rapidly moving toward addressing other challenges that limit NK cell therapeutics and the goal to treat solid tumors. This review describes the state of therapeutic NK cell targeting of tumors; discusses the challenges that need to be addressed before NK cells can be applied as a wide-ranging treatment for cancer; and points to some of the innovations that are being developed to surmount these challenges. Suppressive cells in the tumor microenvironment pose a direct threat to therapeutic NK cells, through presentation of inhibitory ligands and secretion of suppressive cytokines and metabolites. The nutrient- and oxygen-starved conditions under which NK cells must function necessitate an understanding of therapeutic NK cell metabolism that is still emerging. Prior to these challenges, NK cells must find their way into and persist in the tumor itself. Finally, the desirability of a ‘single-shot’ NK cell treatment and the problems and benefits of a short-lived rejection-prone NK cellular product are discussed.

## Introduction

Immunotherapy seeks to enhance the immune response to cancer, harnessing the penetrating and targeted responses of the immune system to bring about elimination of cancer cells with limited side effects. Immune checkpoint blockade (ICB) was an important breakthrough, first approved by the US Food and Drug Administration in 2011. This was followed by chimeric antigen receptor (CAR)-T cells approved in 2017. These two therapies have the potential to cure certain cancers, with ICB demonstrating durable clinical responses in melanoma, lung cancer and other malignancies; and CAR-T cells producing durable responses in clinical trials against leukemia and lymphoma. However, there are drawbacks to these therapies. ICB benefits only a fraction of patients, underserving those with a low mutational burden; and with the remainder of patients at risk of immune-related adverse events. Despite impressive complete response rates for CAR-T cells (83% remission rate in the first clinical trial of Novartis’ Kymriah), cytokine release syndrome and neurological toxicities are frequent complications that have led to comas and death in trials of CAR-T cells. Moreover, this individualized therapy is costly, time-consuming and has yet to show promising responses in solid tumors.

Natural killer (NK) cells have great potential for cancer immunotherapy, because (1) they can recognize mutated cells through a wide array of innate receptors; (2) they kill tumor cells directly and enhance broader immune responses through cytokine production; (3) they can be safely infused into donors with no graft-versus-host disease; and (4) they are amenable to genetic manipulation [1, 2]. Named for their intrinsic capacity to kill cancerous cells, NK cells rely on germline-encoded receptors to detect diseased cells. Inhibitory receptors prevent killing of healthy cells that express human leukocyte class I (HLA-I), while activating receptors trigger cytotoxicity of cells expressing stress proteins. Moreover, NK cells are ‘educated’ by their host environment. If the host lacks ligands for particular inhibitory receptors, those NK cells fail to reach functional maturation and are considered ‘uneducated’ or ‘unlicensed’—unable to respond to many activating signals, whereas the presence of inhibitory ligands allows them to respond to stress proteins. Cancerous cells that downregulate HLA-I and escape the adaptive immune response trigger the ‘missing-self’ response in NK cells—activation in the absence of HLA-I-mediated inhibition [3]. A variety of therapies have been developed to harness the power of these innate immune cells. Cytokines have shown success in expanding and activating this specific subset; antibodies and small molecules have been designed to increase cellular cytotoxicity; and more recently, infusions of cell products have been of increasing interest to the research community.

## Adoptive transfer of NK cells

Various sources of NK cells for cellular immunotherapy are being developed. Transfer of cytokine-activated autologous NK cells showed poor responses to metastatic cancers in early clinical trials [4, 5], whereas mismatched inhibitory receptors and ligands from allogeneic NK cells showed greater clinical responses in hematopoietic stem cell transplantation [6] and adoptive transfer of NK cells [7]. There have been hundreds of allogenic adoptive NK cell transplants performed over the last 16 years, which have repeatedly demonstrated low risk of graft-versus-host disease, cytokine release syndrome and other severe immune-related adverse events [7–12]. To overcome limiting numbers of NK cells from individual donors, strategies to massively expand NK cells are in development [13]. Theoretically, this allows treatment of thousands of patients from a single source. Peripheral blood [14] and umbilical cord blood [15] have both been used as an initial source of NK cells for expansion. Protocols also exist for the development of large numbers of NK cells from stem cells. The source of these stem cells can vary from umbilical cord blood [16] and embryonic stem cells [17] to induced pluripotent stem cells (iPSC) [18]. A third approach uses transformed NK cells (the cell line, NK-92) that can be irradiated to prevent proliferation prior to transfer into patients [19]. These expansion protocols allow for a dosing schedule with multiple infusions of relatively short-lived cells. These allogeneic cell products can be prepared fresh or stored frozen until needed, providing ‘off-the-shelf’ flexibility.

The potential benefits of NK cell therapy are many, but challenges remain before this therapy can benefit patients with a wide range of different cancers. This review summarizes the barriers that stand in the way of broad application of NK cell therapy and how current research is seeking to overcome those barriers.

## Ignorance of tumor

‘Ignorance’ of tumor cells or a failure to recognize malignant cells results in many tumor lines being resistant to NK cell cytotoxicity in vitro. Several therapeutic approaches have been taken to overcome this ignorance and achieve superior clinical responses in vivo. Targeting tumor antigens with antibodies is a well-established field exploiting the overexpression of certain antigens on tumors and not on healthy cells. NK cells express FcγRIIIa (CD16A, referred to as CD16) which binds the Fc portion of antibodies. Triggering of the receptor by antibodies coating diseased cells results in antibody-dependent cellular cytotoxicity (ADCC). CD16 binds to both endogenous and therapeutic antibodies, allowing it to respond to therapeutic interventions. As of 2021, more than 40 monoclonal antibodies have been approved in the USA for the treatment of cancer (antibodysociety.org). Pairing NK cellular therapy with existing monoclonals thus provides a wide range of applications for a single cellular product. Alterations in the design of human monoclonals, including glycosylation [20], particularly afucosylation [21], can further improve the killing dynamics of NK cells. Design alterations to CD16 are also being tested in preclinical models. A fusion of extracellular CD64 with intracellular CD16 was created as a high-affinity alternative to CD16 [22]. It is being explored as a means to preload therapeutic antibodies onto NK cells prior to infusion into a patient. CD16 is normally clipped from the cell surface following NK cell activation, which improves NK cell detachment from tumor cells and serial killing in vitro [23]. A single substitution in CD16 (S197P) can prevent clipping [24] and has shown improvements in ADCC in some preclinical models, but neutral impact in other studies (reviewed in [25]). It is not known whether any deficit in serial killing will be relevant therapeutically for a product that can be dosed repeatedly, but this alteration does allow retention of CD16 surface expression by highly activated cells. Phase I clinical trials are testing the safety of iPSC-derived NK cells bearing unclippable CD16, both in combination with Enoblituzumab (NCT04630769); or with additional cellular modifications, such as a CAR (NCT04555811), or metabolic and cytokine adaptions (NCT04614636). Promising results from a phase II clinical trial of INCB7839, an inhibitor of ADAM17—the enzyme that cleaves CD16 from the cell surface—has shown safety and suggests efficacy in combination with Rituximab against diffuse large B cell lymphoma [26].

The above-mentioned CAR-NK cell is an example of genetic manipulations beyond that of CD16 with the goal of increasing tumor targeting. CARs are of interest because of the success they have shown in the T cell field [27] and the promise they are starting to show in NK cell immunotherapy [8]. Tumor cells can also be targeted through their death receptors [28]. Recently, a variant of TRAIL with specificity for TRAILR2 was shown to increase killing of multiple myeloma cell lines by an NK cell line in vitro [29]. Furthermore, genes can be removed from NK cellular products that would otherwise impair their responses. CD38 is overexpressed by multiple myeloma and targeting this antigen is under investigation as a treatment for this and other malignancies, but NK cells express CD38, especially when expanded ex vivo. Preclinical studies have shown that knocking out CD38 can reduce NK cell fratricide in the presence of the anti-CD38 monoclonal antibody daratumamab [30]. iPSC-derived NK cells that have unclippable CD16 and knockout of CD38 are entering clinical trials for the treatment of acute myeloid leukemia in concert with daratumumab (NCT04714372).

Cell therapies under development will also potentially benefit from novel biologics that enhance targeting of tumors. These are designed to bind CD16 and other activating receptors found on NK cells. Single chain variable fragment (scFv) domains of antibodies targeting CD16 have been incorporated into bispecific (tetravalent) [31] and trispecific molecules (NCT03214666) [32], but some of the latest configurations incorporate single-domain anti-CD16 moieties [33, 34]. In the case of trispecific killer engagers (TriKE®), this has been shown to bind with greater affinity to CD16 than the scFv [34]. Biologics targeting other activating receptors on NK cells, including NKG2D, NKp30 and NKp46 [35], are being developed because of the potential loss of CD16 in the tumor microenvironment and the shared expression of CD16A/B on other innate immune cells. A killer engager targeting activating receptor NKG2C was also recently developed, aiming to capitalize on the desirable attributes of NKG2C-expressing adaptive NK cells [36]. Trifunctional natural killer cell engagers that target activating receptor NKp46 in tandem with an Fc domain that engages CD16 have shown enhanced activation compared to bispecific molecules [37]. Several of these biologics incorporate cytokine variants, providing a boost in activation in addition to the survival and persistence benefits discussed later.

Thus, thanks to continued efforts in this area of research, ignorance of tumor is not the challenge to NK cell immunotherapy that it once was. Experience from the CAR-T cell field cautions against targeting single tumor antigens and so, moving forward, this field is combining many of these CAR, therapeutic antibodies, biologics, enhanced ADCC strategies and death ligands to prevent tumor escape. Most of these approaches are at the preclinical stage of development, but clinical trials targeting multiple antigens are already launching, most prominently CD19 and CD20 for the treatment of leukemia and lymphoma (NCT04245722, NCT04555811, NCT03824964). Antigen targeting and combined strong activating signals can lessen the impact of an immunosuppressive environment, but addressing these inhibitory factors directly is the approach discussed in the next section.

## Suppressive cells

The cellular make-up of the tumor microenvironment (TME) consists of malignant cells, that themselves can secrete, present or withhold factors in order to suppress the immune response, but stromal cells and immune cells can also suppress NK cell anti-cancer activity. Together these present a challenging microenvironment for a therapeutic NK cell.

Inhibitory checkpoint ligands are shared by many suppressive cells in the TME, thus targeting them is a broad mechanism to release suppression from NK cell therapies. Several clinical trials are combining adoptive transfer of NK cells with checkpoint blockade: in advanced solid tumors (NCT03841110; NCT03941262), Merkel cell carcinoma (NCT03853317), biliary tract cancer (NCT03937895) and head and neck cancer (NCT04290546). Checkpoint blockade affects the immune cell milieu, but can also have direct effects on, or be mediated by NK cells. NK cells express inhibitory receptor PD1 at low levels which can be upregulated in vivo [38]. In a xenograft mouse model of ovarian cancer, knocking out the gene encoding PD1, *PDCD1*, in adoptive NK cells, resulted in better tumor control and survival [39]. Furthermore, checkpoint antibodies can drive ADCC of tumor cells or suppressive cells in the TME. Of the trials listed above, NCT03853317 is a phase II trial utilizing an IgG1 isoform of anti-PDL1 antibody, avelumab, which drives ADCC, and is being tested in combination with allogeneic NK cells and N-803, an IL-15 receptor agonist. Similarly, the probability of success of anti-CTLA-4 treatment ipilimumab in melanoma patients increases when high-affinity polymorphisms in CD16 (V158F) are present, suggesting that NK cells and monocytes bearing this receptor are involved in the depletion of suppressive Tregs at the tumor site [40]. New checkpoint antibodies to directly target NK cells are also under development. Lirilumab is an anti-killer cell Immunoglobulin-like Receptor checkpoint antibody that has shown safety [41], but very limited efficacy in vivo [42, 43]. The variable expression of KIR on allogeneic NK cells makes it a less tempting prospect than targeting the broadly expressed NKG2A for combination with NK cell therapy [44]. Anti-NKG2A checkpoint antibody, monalizumab, releases inhibition from both NK cells and T cells [45] and is being tested in combination with cetuximab in a Phase III trial against squamous cell carcinoma of the head and neck (NCT04590963).

Focusing on suppressive molecules within the TME, indoleamine-2,3-dioxygenase is currently the target of a number of clinical trials [46]. Among broad immunosuppressive effects in the TME, kynurenine produced by idoleamine-2,3-dioxygenase activity directly inhibits NK cell cytotoxicity [47] and proliferation [48]. Tumor cells can also secrete prostaglandin E2, which induces myeloid-derived suppressor cells (MDSC) [49], suppresses NK cell function [50] and suppresses NK cell-mediated recruitment of conventional dendritic cells to the TME [51]. In the latter animal model, dendritic cells normally drive protective T cell responses in the TME, a process disrupted by prostaglandin E2 secretion. Arginase is another secreted molecule in the TME that can suppress NK cell responses. NK cells are less sensitive than T cells to the depletion of arginine by arginase, but when sufficiently low quantities of L-arginine are available, both interferon-γ (IFNγ) production and NK cell proliferation are impaired [52]. An inhibitor of arginase CB-1158 reduced lung metastases when combined with adoptive transfer of NK cells in a mouse model, leading to clinical trials of this agent in combination with therapeutic antibodies (NCT03837509, NCT02903914, NCT03910530) or chemotherapy (NCT03314935) [53].

Blocking the conversion of immunogenic adenosine triphosphate into immunosuppressive adenosine by ectoenzymes CD39 and CD73 is another aspect of TME suppression that is under clinical investigation [54, 55]. Most studies of the direct effects of adenosine on NK cells have been limited to mice, but human NK cells express adenosine 2A receptors [56], suggesting that human NK cells are also sensitive to extracellular adenosine. Hypoxia is a strong driver of adenosine production and hyperbaric oxygen administration to mice was shown to act upstream of adenosine suppression, suggesting reversing tumor hypoxia can circumvent some broader immunosuppressive effects. Indeed, in mouse models of metastasis, supplemental oxygen was able to prevent metastases, but only when NK cells were present [57]. Antibodies that block the activity of CD39 and CD73 can additionally drive human NK cell ADCC of tumors overexpressing these molecules [58, 59], suggesting potential synergy between NK cell therapies and trials disrupting adenosine production.

TGFβ is one of the most potent immunosuppressive mechanisms of the TME. TGFβ ‘traps’ that bind TGFβ in the tumor environment and prevent it triggering immune cells have shown promising preclinical data and a good safety profile in a phase I clinical trial [60]. Fusions of TGFβ traps and immunostimulatory IL15 are also in development [61]. Moreover, direct alteration of NK cellular products can bypass the release or presentation of suppressive molecules. Dominant negative TGFβR2 [62, 63] rendered NK cell products resistant to the suppressive effects of TGFβ.

Of suppressive immune cells within the TME, MDSC, tumor-associated macrophages (TAM), regulatory T cells (Treg) and neutrophils have some of the best characterized interactions with NK cells. Their presence within tumors is associated with a poor prognosis across a wide range of tumor types [64]. MDSCs are a heterogeneous group of myeloid cells that can be either granulocytic or monocytic in phenotype. In humans MDSC generally have low expression of HLA-DR [65], whereas TAMs have a mature myeloid cell phenotype. They are best characterized for their ability to suppress T cell responses, but can also suppress NK cell cytotoxicity through inhibitory receptor triggering [66], or through release of TGFβ1 [67, 68], nitric oxide [69] or reactive oxygen species [67, 70, 71].

A few approaches have been taken to use NK cells to directly eliminate suppressive cells. Bispecific killer engagers(BiKEs) and TriKEs that bind CD33 target NK cells toward acute myeloid leukemia, but can also induce NK cells to kill MDSC and prevents their suppressive effects in vitro [72, 73]. A chimeric receptor consisting of extracellular NKG2D fused to CD3ζ intracellular domain can similarly target NK cells toward MDSC that overexpress a number of NKG2D ligands [74]. In patients with multiple myeloma, daratumumab treatment can deplete, not only the cancer, but also MDSC, Treg and regulatory B cells that express CD38 [75]. Strategies targeting CD38, which include daratumumab and CAR-NK cells [76], could therefore enhance NK cell immunotherapy by altering the suppressive TME. This has been demonstrated with immunosuppressive stromal cells and CD38-targeted CAR-NK cells in vitro [29]. Less research has been done to determine how key receptor interactions might be targeted to disrupt the suppressive cross talk between myeloid cells and NK cells. Investigations of NK-myeloid interactions in liver cancer showed myeloid cells drove NK cell exhaustion via CD48-2B4 interactions. Blocking 2B4 was thus able to prevent TAM-induced NK cell apoptosis in vitro [77].

Tregs can suppress NK cell proliferation and activation through competition for IL-2 in mouse models [78]. They also suppress NK cell reconstitution in relapsed acute myeloid leukemia patients, but delivery of an IL-2-diptheria toxin fusion prior to NK cell infusion, increased the rate of NK cell reconstitution and improved complete remission rates in a phase I clinical trial [9]. At least part of the suppressive effects on NK cell expansion in this study was attributed to the competition for IL-2, since NK cells proliferated in vitro in the presence of Treg with the addition of IL-15, but not IL-2. Tregs express a high-affinity receptor for IL-2, IL2Rα/CD25, that outcompetes NK cells for this resource. Therapeutic treatment with IL-15 homologs have been developed to stimulate the beneficial cytotoxic NK and T cell functions, bypassing this suppressive effect of Tregs and the dose limiting toxicities that can arise through IL-2.

Tregs can also suppress NK cell function through TGFβ surface presentation [79] and promote T cell exhaustion via IL-10 and *PRDM1* in mouse models of cancer [80]. Given that Blimp1, encoded by *PRDM1*, is a negative regulator of NK cell function [81] it can be hypothesized that tumor-associated Tregs might also induce NK cell suppression via this mechanism. Suppression of IL-10 improves checkpoint blockade and reduces T cell exhaustion in mouse models of CLL [82], but counterintuitively, pegylated IL-10 can also have potent immune stimulatory effects on CD8 T cells and is being explored as an anti-cancer therapy [83]. Therefore, while IL-10 can suppress NK cell functions, therapeutic disruption of IL-10 signaling for enhancement of adoptive NK cell transfer must be carefully considered in the particular cancer context, whereas disruption of TGFβ is more likely to have broad beneficial effects on adoptive NK cell therapy.

There are global associations of poor cancer prognosis with the circulating neutrophil-to-leukocyte ratio. Research in the field of T cell biology has shown that RANK signaling invites in neutrophils that prevents CD8 T cell accumulation [84]. In mice, Micaeli et al. have shown that granulocytic myeloid regulatory cells/tumor-associated neutrophils are capable of inducing apoptosis of non-activated CD8 T cells and subsequently impair anti-tumor responses in vivo [85]. Release of extracellular traps by neutrophils and granulocytic MDSC have been shown to shield tumor cells from NK cell cytotoxicity [86]. Mice treated with GSK484 that blocks the release of extracellular traps had fewer lung metastases dependent upon the presence of NK cells, suggesting that preventing recruitment of neutrophils to the TME and their release of extracellular traps has the potential to improve NK cell immunotherapy. Thus, with several approaches addressing the impact of suppressive cells on NK cell function, this leaves challenges inherent to the TME, such as scarcity of nutrients, to be overcome.

## Metabolic limitations

Hanahan and Weinberg included reprogramming of energy metabolism in their updated list of cancer hallmarks, published in 2011 [87]. Cancer cells exhibit Warburg metabolism: aerobic glycolysis that provides metabolic intermediates required for increased proliferation. Drawing parallels for NK cells from other lymphocytes, it has been proposed that competition for glucose is a limiting factor that prevents T cell immune responses [88]. Recent evidence, also from T cells, suggests that rather than competition, the glucose limitation may be a cell intrinsic limitation brought about by metabolic programming dictated by the TME [89]. ‘Mechanistic target of rapamycin’ (mTOR), an important nutrient and metabolic sensor, contributed to this reprogramming. Their data suggest glucose is not limiting in the tumor microenvironment, although tumor cells dominate glutamine uptake. In line with this, concentrations of glucose detected in the bone marrow of multiple myeloma patients (500 mg/L) failed to show any impairment of human NK cell cytotoxicity in vitro [90]. An analysis of intracellular metabolites in licensed and unlicensed expanded NK cells revealed lower glutamate concentrations in licensed NK cells, suggesting they may rely more on glutaminolysis than unlicensed cells [91]. In mice it has been shown the glutamine restriction limits cMyc availability and consequently impairs NK cell cytotoxicity [92]. However, in vitro experiments with human NK cells suggest they are relatively resistant to metabolic restriction, continuing ATP production at concentrations of glucose and glutamine that impair CD8 T cells [93].

It is known that NK cells increase both glycolysis and oxidative phosphorylation upon cytokine activation [94] or activating receptor triggering [95], with both metabolic processes required for IFNγ production. Expansion protocols dramatically increase glycolysis and glycolytic capacity, with upregulation of PKM and GLUT8, and retention of GLUT1, GLUT2 and mTOR [91]. While oxidative phosphorylation is required for primary NK cell cytotoxicity [94], expanded NK cells depend upon glycolysis, with no impact on cytotoxicity when oxidative phosphorylation is blocked [95]. In contrast to this latter study, Schafer et al. observed almost complete inhibition of cytotoxicity when mitochondrial respiration was inhibited, but only for unlicensed NK cells with very modest decreases in cytotoxicity of licensed NK cells [91]. Similarly, in freshly isolated cells, glucose uptake is high in KIR-educated or NKG2A + cells compared to uneducated NK cells, with overnight culture in glucose-free media decreasing degranulation of uneducated NK cells, while educated NK cells are unaffected [96]. These data suggest that educating receptors on expanded NK cells may facilitate greater cytotoxicity within the hypoxic tumor microenvironment. However, maintaining IFNγ production, which is dependent on oxidative phosphorylation, requires further adaptation.

mTOR, the catalytic subunit of both mTORC1 and mTORC2 complexes, is an important effector of metabolic change in NK cells, but is also a target of several tumor therapies. In freshly isolated NK cells, mTORC1 signaling is not required for short-term cytotoxicity assays. IL-2 upregulation of glycolytic machinery is sensitive to rapamycin, but IL-12/IL-15-mediated upregulation is unaffected. In parallel, IFNγ production is sensitive to rapamycin in IL-2-stimulated, but not IL-12/IL-15-stimulated cells [94]. Inhibitors of mTORC1 used to treat breast cancer resulted in fewer circulating NK cells in a prospective study of sixty women [97], so consideration must be taken when combining NK cell therapy with chemotherapy. Short-lived effector cells maintained by IL-15 may be a better choice for combination therapy. TGFβ produced in the TME can also drive metabolic defects [98], which should be relieved by the mechanisms targeting TGFβ described above, as well as pathways that increase mTORC1/GAPDH/SLC2A1 in therapeutic NK cells(reviewed in [99]). IL-15 and IL-2 signaling are common ways to boost mTORC1 signaling, and many molecular mimics of these molecules have been designed in order enhance therapeutic NK cell responses.

Less information is available on how metabolic activity of the tumor might influence NK cell responses. Tumor glycolysis can drive lactic acid buildup in the TME, and 15 mM lactic acid treatment of NK cells ex vivo has been shown to completely abolish IFNγ production [100]. These authors found that manipulating LDHA levels in a mouse model of melanoma results in more infiltrating NK cells and greater expression of IFNγ and granzyme B and greater tumor control in low-LDHA tumors compared to controls. Several approaches to manipulating tumor cell metabolism are being taken in the clinic, but exactly how these might impact therapeutic NK cells is yet to be determined.

## Competition for oxygen

Hypoxia induces mTOR signaling and subsequent mitochondrial fragmentation that limits cytotoxic responses of tumor-infiltrating NK cells isolated from patients with liver cancer, or peripheral blood NK cells exposed to hypoxia ex vivo [101]. In this context, 3–7 day treatment with rapamycin during exposure to hypoxia prevented mitochondrial fragmentation. Mitochondrial fragmentation was associated with a drop in maximal oxidative respiration, which could also be prevented by inhibiting mitochondrial fission by knocking out Drp1 or pre-treating with mdivi-1. Impaired cytotoxicity and tumor control by NK cells pre-exposed to hypoxia could similarly be prevented by pre-treatment with mdivi-1 in a xenograft mouse model. These data suggest mitochondrial fragmentation may be an important consideration in NK cell therapy; however, since expanded NK cell cytotoxicity is largely independent of oxidative phosphorylation, as described above, these concerns may already be addressed by glycolytic reprogramming induced by expansion protocols. Maintenance of IFNγ production in the hypoxic environment, however, needs to be addressed, since this process is dependent on oxidative phosphorylation, even in expanded NK cells.

Hypoxia-inducible factor 1α (HIF1α) is a transcription factor downstream of mTOR activation. Subcutaneous mouse models of lymphoma and lung cancer have shown that deletion of HIF1α in NK cells delayed tumor growth [102]. The RMA-S subcutaneous model used in this study is known to be sensitive to IFNγ and the authors found that single cell RNA sequencing identified a HIF1α−/− population of NK cells with increased IFNγ responses. In parallel, human NK cells, isolated and sequenced from non-small cell lung cancer patients, had higher HIF1a expression and lower IFNγ expression when NK cells were isolated from within the tumor compared to the peritumor region. After exposure to hypoxia ex vivo, HIF1α−/− NK cells had higher levels of oxidative phosphorylation and IFNγ production following IL-18 treatment. How HIF1α knockout will affect expanded NK cells is yet to be determined, but one of the hoped for benefits would be a greater capacity for oxidative phosphorylation and IFNγ production.

A greater understanding of these pathways will lead to more options for adapting therapeutic NK cells. In 2017, researchers showed that knocking out HIF1α in tumor-infiltrating T cells increases peroxisome proliferator-activated receptor-α (PPAR-α) expression in these cells, a transcription factor which promotes fatty acid catabolism. Switching to fatty acid catabolism, through HIF1α−/− or PPAR-α agonists, maintains CD8 T cell responses to melanoma in mouse models where oxygen is limiting [103]. By extrapolation, these data suggest that PPAR-α agonists might also promote stronger NK cell responses where oxygen is limiting.

## Tissue access

Genetic overexpression of chemokine receptors to increase NK cell trafficking to the TME is a principle explored for a range of chemokine receptors in preclinical models (CCR5, CCR7, CXCR1/2, CXCR4, CX3CR1, reviewed in [104]). These experiments have demonstrated improved infiltration of NK cells into solid tumors, delays in tumor growth and improved survival in particular cancer models. Genetic alteration is not the only means of improving NK cell homing. Expanded NK cells co-cultured for an hour with CCR7-expressing feeder cells, transiently acquired CCR7 by trogocytosis which increased the number of NK cells entering the lymph node in nude mice [105]. The presence of chemokine receptors on NK cells is an important consideration, given expanded NK cells do not necessarily have the same chemokine receptor expression profile as endogenous NK cells.

One of the key challenges is that chemokines vary across tumor types, so genetic alterations of NK cells may have to be tailored to particular tumors. Creative ways have been found to change the TME, rather than the NK cells, to increase NK cell recruitment. GPI-anchored chemokines that insert into tumor membranes increase NK cell recruitment when injected into solid tumors in mice [106]. More generally, access to the TME can be the result of metabolic limitations and suppressive cell interactions. Blocking IL-8 signaling is being approached as a means to prevent suppressive MDSC and neutrophil recruitment to the TME. Moreover, in a murine model of colon cancer described above, treatment with an inhibitor of the mTORC1 pathway (rapamycin) or an inhibitor of glutamine uptake (V9302) reduced NK cell tumor infiltrates [89]. Targeting metabolic and immunosuppressive challenges may thus also improve NK cell access to tissues. Here there may be parallels with T cell biology; for example, in urothelial cancer, it was found that TGFβ signaling in stroma cells predicted clinical response to checkpoint blockade. High levels of TGFβ signaling were associated with exclusion of CD8 T cells from within the tumor. In a mouse model, tumor control could be initiated by blocking TGFβ signaling which reprogrammed stromal fibroblasts and increased CD8 T cell penetrance in the presence of checkpoint blockade [107]. In line with this, inhibition of the TGFβ receptor kinase by LY2157299 improved infiltration of adoptive NK cells in a mouse liver metastases model of colon cancer, reducing the tumor burden [108].

Suppressive elements of the TME can also be used as a homing device and activator for NK cell products. The extracellular domain of TGFβRII fused to the intracellular domain of NKG2D when expressed in NK92 showed subtle improvement in homing to hepatocellular tumors than NK92 controls in a xenograft model of liver cancer [109]. A clinical trial is also recruiting to test RO7284755, a bispecific molecule that blocks PD1 and delivers IL-2, which aims to stimulate NK cells and T cells specifically within the TME (NCT04303858).

Finally, allowing NK cells to access immune privileged sites can be targeted by specific nanoparticles. Polymers that are ferried across the blood–brain barrier into an otherwise immune privileged site allowed NK cell recruitment into the TME and improved survival in mouse models of glioblastoma [110].

## Persistence/survival

Autologous CAR-T cell therapies require administration of a single dose for prolonged therapeutic effect. ‘Off-the-shelf’ NK cell therapies can be safely administered in large doses multiple times, mostly bypassing the problem of persistence that was initially encountered by poor NK cell engraftment without lymphodepletion [7]. However, as an allogeneic product, the cells are susceptible to rejection by the host. In most cases, circulating cells are only detected by flow cytometry for a few weeks after dosing [7–9]. Short-term gains in persistence that reduce the need for frequent doses can be made through cytokine signaling that enhances NK cell survival. This can come in the form of independently administered cytokine analogs, such as recombinant IL-15, heterodimeric IL-15-IL15Ra or N-803 ‘superagonist’ comprising IL-15 and bivalent IL15Ra Fc fusion [111]. Alternatively, IL-15 transgene expression has facilitated increased NK cell persistence in tissues and tumor control in xenogenic mouse models of lymphoma [112]. Transgene DNA from this CAR-NK cell product was detected in patients a year after transfusion [8], suggesting longer-term persistence in patients may be possible with the right modifications under the right conditions. A third mechanism entails expression of membrane-bound cytokines in NK cell products. A ‘receptor fusion’ of IL-15-IL15Ra combined with a knockout of CD38 results in iPSC-derived NK cells with features of adaptive NK cells, including greater resistance to oxidative stress-induced apoptosis and enhanced survival in vivo [113]. Knocking out negative regulator of cytokine signaling CIS can further boost NK cell responses to cytokines [114], enhancing metabolic function, increasing persistence and tumor control in preclinical models of iPSC-derived and cord blood-derived NK cells [115, 116]. However, these approaches do not address the fundamental hurdle of rejection.

In pursuit of allogeneic ‘off-the-shelf’ products, the T cell field has started to develop rejection-resistant cells that lack classical HLA class I expression. However, if repeat dosing of NK cells were to be abandoned in favor of long-term persistence, new challenges might arise. For example, hypoxia has been shown to impair the transformation of CAR-T cells into effector memory cells [117], which is not currently a challenge for short-lived therapeutic NK cells. Loss of CAR expression and exhaustion are similar challenges for T cell therapy that are less impactful for NK cell therapy with current dosing schedules. In terms of long-term metabolic fitness, excessive antigenic T cell stimulation results in high intracellular calcium levels even at rest and rapid induction of mitochondrial stress. This limits T cell capacity for oxidative phosphorylation and subsequent function, despite enhanced glycolytic capacity and large mitochondrial mass. Applying anti-oxidants can restore T cell effector functions and tumor immunity, but only when oxygen is present [118]. Another study of chronic T cell stimulation, this time combined with hypoxia, found reactive oxygen species and chronic NFAT1 signaling drove an exhausted phenotype of T cells. Knocking out *PRDM1*, which suppressed PPAR-γ co-activator (PGC) 1α, which upregulates antioxidant enzymes, increased mitochondrial mass and IFNγ production of tumor-infiltrating T cells exposed to chronic hypoxic stimulation ex vivo. In line with this, overexpression of *Gpx1,* an antioxidant target of PGC1α, reduced reactive oxygen species within tumor-infiltrating T cells and boosted IFNγ production [119]. If long-term persistence is to be the goal in NK cell therapy, consideration must be given to avoiding exhaustion brought on by chronic stimulation [120, 121]. Inhibitory checkpoint receptor expression is just one aspect of dysfunctional NK cell states. Exhaustion, anergy and senescence are distinct dysfunctional states in T cells, but their relevance to NK cell biology is still being defined [122]. While the current administration of multiple doses has its drawbacks in cost and inconvenience, it does allow for treatment discontinuation for patients with side effects, avoiding the need for suicide genes that abruptly terminate a dangerous T cell response. The disappearance of infused cells from circulation after a few weeks also means that side effects—such as the loss of normal B cells in CAR19-T cell therapy—are also potentially less permanent.

## In summary: NK cell therapy as part of a coordinated response

**Fig. 1:**
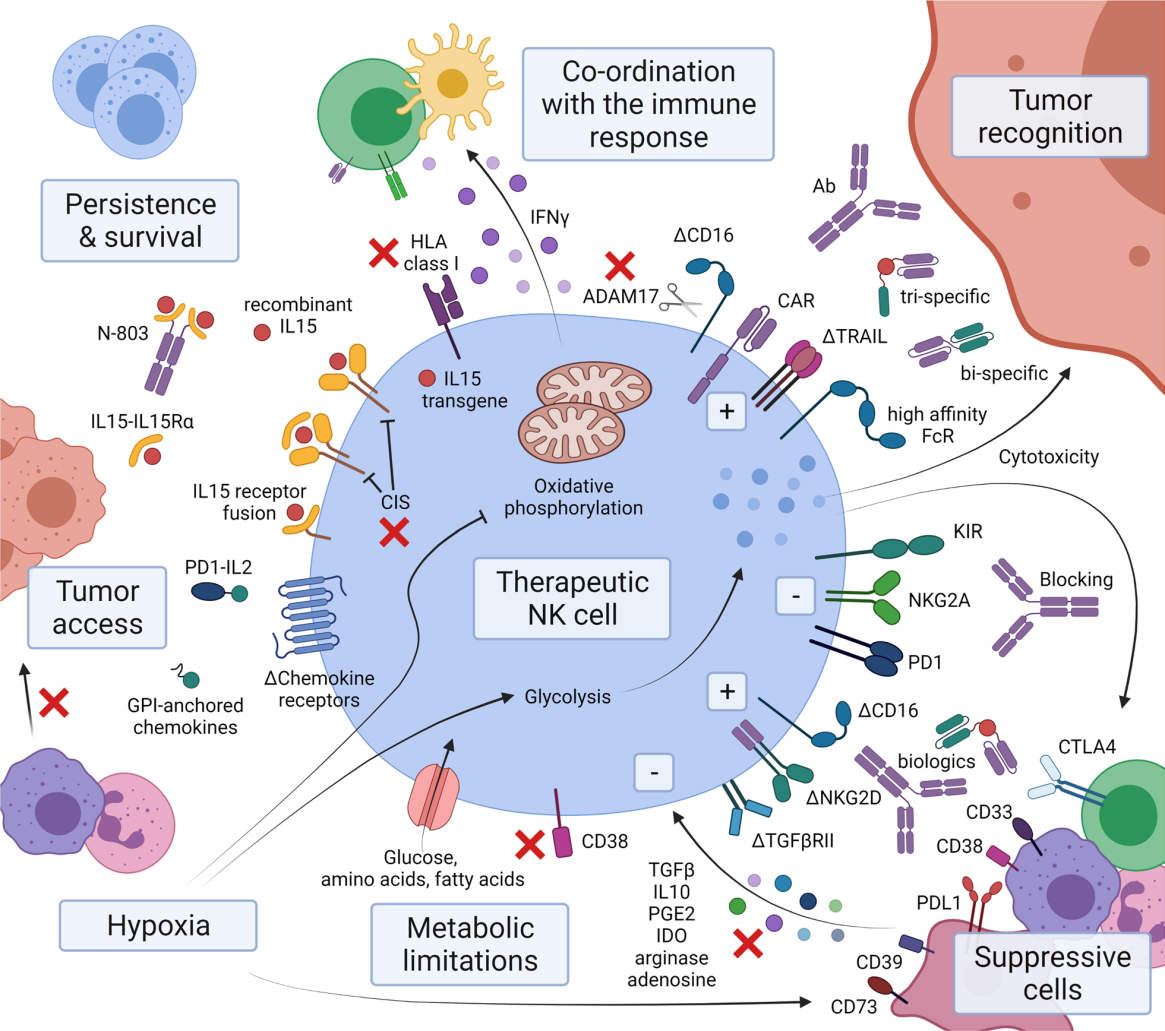
Strategies to address the challenges to NK cell therapy. The complex tumor microenvironment poses several challenges to successful NK cell therapy. This schematic highlights some of the innovations covered in this review that are addressing these challenges in preclinical and clinical studies. Δ = reengineered protein, red ‘x’ = targeted for blocking, prevention or deletion. Created with BioRender.com

Overall ‘off-the-shelf’ expanded NK cell products entering clinical trials are adaptable and highly specific when it comes to tumor recognition. The suppressive TME still poses a considerable challenge in terms of NK cells gaining access and sustaining function; however, several adaptions have been tested pre-clinically to overcome some of the most pressing challenges (Fig. 1). Metabolically, expanded NK cells are distinct from endogenous cells. Fractured mitochondria and reactive oxygen species accumulation seen in endogenous NK cells and T cells are not necessarily the same problems faced by NK cell products, with these distinctions requiring further investigation. There is also an important distinction as to which NK cell functions are sensitive to metabolic challenges. Cytotoxicity is maintained by glycolysis, but cytokine production requires oxidative phosphorylation, while proliferative capacity may not be as relevant for a short-lived cellular product. Prioritizing adaptations will require consideration of what functions are most desirable in an NK cellular product. Direct cytotoxicity of malignant cells has obvious value, but IFNγ production and coordination of a long-term immune response has shown considerable value in animal models and human correlative studies.

NK cells are fundamentally innate, with an ideal treatment outcome being the resetting of the immune response to facilitate long-term adaptive control without the need for repeated treatment. There is preclinical evidence to suggest this is possible. NK cells, designed to eliminate MDSC, facilitate increased infiltration of subsequently infused CAR-T cells into the TME and improve mouse survival [74]. Mouse models have shown NK cells recruit conventional dendritic cells to the TME, enhancing immune control [51] and NK cell production of IFNγ boosts T cell activity, predicting greater response to ICB [123]. Indeed, iPSC-derived therapeutic NK cells combined with systemic T cell infusions increase T cell trafficking to the peritoneal cavity in a peritoneal model of ovarian cancer, where anti-tumor activity is further enhanced in this ‘hot’ tumor microenvironment by ICB [124]. There may be many benefits to combining therapies to coordinate the immune response as we learn more. Adaptations must be made to allow NK cells to work alongside current standard of care interventions. One final example of this comes from the field of γδ T cells, which were rendered resistant to DNA damage induced by temozolomide through forced expression of P140KMGMT [125]. One of the key benefits of such a safe, adaptable therapy, poised to shape a complex immune response, is that, however, this field progresses, these cells are likely to combine well with other therapies and innovations.

## Data Availability

Not applicable.

## Abbreviations

ADCC: Antibody-dependent cellular cytotoxicity
BiKE: Bispecific killer engager
CAR: Chimeric antigen receptor
Fv: Variable fragment (of immunoglobulin)
HIF1α: Hypoxia-inducible factor 1α
HLA-I: Human leukocyte antigen class I
ICB: Immune checkpoint blockade
IFNγ: Interferon-γ
iPSC: Induced pluripotent stem cells
MDSC: Myeloid-derived suppressor cell
mTOR: Mechanistic target of rapamycin
NK: Natural killer
PGC1α: PPAR-γ co-activator 1α
PPAR-α: Peroxisome proliferator-activated receptor α
scFv: Single chain variable fragment
TAM: Tumor-associated macrophage
TME: Tumor microenvironment
Treg: Regulatory T cell
TriKE: Trispecific killer engager
